# Cancer predisposition signaling in Beckwith-Wiedemann Syndrome drives Wilms tumor development

**DOI:** 10.1038/s41416-023-02538-x

**Published:** 2023-12-23

**Authors:** Snehal Nirgude, Natali S. Sobel Naveh, Sanam L. Kavari, Emily M. Traxler, Jennifer M. Kalish

**Affiliations:** 1 Division of Human Genetics and Center for Childhood Cancer Research, Children’s Hospital of Philadelphia, Philadelphia, PA 19104 USA; 2 Departments of Pediatrics and Genetics, Perelman School of Medicine at the University of Pennsylvania, Philadelphia, PA 19104 USA

**Keywords:** Cancer genomics, Cancer genetics

## Abstract

**Background:**

Wilms tumor (WT) exhibits structural and epigenetic changes at chromosome 11p15, which also cause Beckwith-Wiedemann Syndrome (BWS). Children diagnosed with BWS have increased risk for WT. The aim of this study is to identify the molecular signaling signatures in BWS driving these tumors.

**Methods:**

We performed whole exome sequencing, methylation array analysis, and gene expression analysis on BWS-WT samples. Our data were compared to publicly available nonBWS data. We categorized WT from BWS and nonBWS patients by assessment of 11p15 methylation status and defined 5 groups– control kidney, BWS-nontumor kidney, BWS-WT, normal-11p15 nonBWS-WT, altered-11p15 nonBWS-WT.

**Results:**

BWS-WT samples showed single nucleotide variants in *BCORL1*, *ASXL1*, *ATM* and *AXL* but absence of recurrent gene mutations associated with sporadic WT. We defined a narrow methylation range stratifying nonBWS-WT samples. BWS-WT and altered-11p15 nonBWS-WT showed enrichment of common and unique molecular signatures based on global differential methylation and gene expression analysis. CTNNB1 overexpression and broad range of interactions were seen in the BWS-WT interactome study.

**Conclusion:**

While WT predisposition in BWS is well-established, as are 11p15 alterations in nonBWS-WT, this study focused on stratifying tumor genomics by 11p15 status. Further investigation of our findings may identify novel therapeutic targets in WT oncogenesis.

## Introduction

Wilms tumor (WT), or nephroblastoma, is the most common pediatric kidney cancer [1]. While modern treatment strategies and methodologies have increased survivability, poor outcomes still occur, particularly in the setting of bilateral, relapsed, and anaplastic tumors. Although healthy children develop WT, children who have cancer predisposition disorders like Denys-Drash syndrome, WAGR syndrome, or Beckwith-Wiedemann syndrome (BWS, OMIM 130650), are more likely to develop WT [2–4]. Denys-Drash syndrome and WAGR syndrome are due to alterations of *Wilms Tumor 1* (*WT1*) [5], and mutations in *tumor protein 53* (*TP53*) [6, 7] and *beta-catenin* (*CTNNB1*) [8, 9] are associated with WT susceptibility.

BWS comprises a range of fetal and neonatal overgrowth characteristics including nephromegaly, urogenital malformations, and development of embryonal tumors, including WT [10–13]. Patients with BWS are at an approximately 800-fold increased relative risk of developing WT; these neoplasms occur at a younger age and at increased incidence, as compared to age-matched, non-syndromic children [12, 14–16]. Due to these factors, tumor screening is recommended for patients with BWS by ultrasonography every three months through the 7th birthday [17].

Loss-of-heterozygosity (LOH) is both a common somatic oncogenic driver within tumors and can be present in the somatic tissues of patients with a mosaic cancer predisposition syndrome like BWS [18]. LOH is reflected in parallel alterations on 11p15 in both BWS and present somatically in at least two thirds of WT [19]. For example, one cause of BWS is mosaic loss of heterozygosity (LOH) at 11p15 caused by paternal uniparental isodisomy (pUPD11) [19], which leads to disruption of an imprinting control region that regulates parent-of-origin-specific gene expression [20]. LOH at chromosome 11p15 is also present in at least 30% of WT and is associated with higher staging classification and a greater risk for relapse [5, 7, 19, 21–26]. LOH or pUPD11 leads to gain of methylation (GOM) at the differentially methylated region (DMR) *H19/IGF2:IG-DMR*, which regulates Imprinting Center 1 (IC1), and causes increased paternal expression of *Insulin-like Growth Factor 2* (*IGF2*) and decreased maternal expression of non-coding RNA *H19* [11]. Additionally, it also leads to loss of methylation (LOM) at the *KNCQ1OT1:TSS-DMR*, which regulates Imprinting Center 2 (IC2), and causes decreased maternally expressed *Potassium Voltage-Gated Channel Subfamily Q member 1 (KCNQ1)* and *Cyclin-Dependent Kinase Inhibitor 1C* (*CDKN1C*) as well as increased expression of non-coding antisense RNA *KCNQ1OT1* [11]. In some tumors, the LOH extends through 11p13 and dysregulates the *WT1* gene [27, 28]. ~30% of WT have isolated IC1 GOM without the LOH [26, 27, 29], which is also a cause of BWS.

Given the propensity of BWS patients to develop WT and the fact that at least two-thirds of WT have alterations at 11p15 (either LOH or IC1 GOM), we stratified patients by 11p15 status to study downstream signaling using a multi-omics approach. In this study, we used DNA methylation arrays, whole exome sequencing, and RNA sequencing to elucidate the cellular signaling pathways involved in BWS-WT development. We compared our BWS cohort with nonBWS-WT, stratified by 11p15 status, to shed light on the genomic, methylomic, and transcriptomic changes that arise because of 11p15 alterations. This experimental design enabled us to define molecular signatures in both BWS-WT and nonBWS-WT. Finally, using a weighted gene co-expression network analysis (WGCNA) approach, we aimed to systematically investigate the pattern of gene associations among samples in both cohorts based on mRNA level in WT.

## Methods

### Patients and samples

Samples and clinical information were collected through the BWS Registry, under the oversight of the Children’s Hospital of Philadelphia (CHOP) Institutional Review Board protocol (IRB 13-010658). In brief, consent was obtained from all patients and/or legal guardians to collect longitudinal clinical information, in addition to samples that became available through clinical care.

During surgical procedures for WT resection, tumor and adjacent kidney samples were collected from patients, then snap frozen in liquid nitrogen and stored at −80 °C. Clinical testing for BWS molecular characterization was performed in blood, non-tumor kidney, and tumor at the University of Pennsylvania Genetic Diagnostic Laboratory, as previously described [30]. We analyzed nine primary BWS-WT samples available to us; six had matched non-tumor kidney samples that were collected.

Three control kidney samples (C1, C2, C3) were collected at autopsy with consent through CHOP Pathology and Laboratory Medicine. Subsequent testing at 11p15 was performed to verify the samples did not demonstrate molecular BWS. Basic information about these samples was provided through an honest-broker. An additional two control kidney samples (C4, C5) were collected in a similar way, and were used in transcriptome analysis to compare to tumor profiles.

### NonBWS-WT data

Non-syndromic data was retrieved from the National Cancer Institute (NCI) Therapeutically Applicable Research To Generate Effective Treatments (TARGET) Initiative [31] and the Gene Expression Omnibus (GEO) accession number GSE110697 (referred to as Murphy et al. data) [32]. For the methylation study, 21 samples were used from validation set of TARGET [31] and 39 samples were used from Murphy et al. [32]. For gene expression data, 21 WT samples were used from the validation set of TARGET data.

Data generated in this study have been deposited in the Database of Genotypes and Phenotypes (dbGAP) of the National Center for Biotechnology Information (United States National Library of Medicine, Bethesda, MD) under accession number phs002769.v1.p1.b. A detailed summary of sample distribution in the study is depicted in Supplementary Fig. 1.

### Genomic DNA isolation

Genomic DNA from kidney samples was isolated using the AllPrep DNA/RNA Micro Kit (QIAGEN) and quantified on Qubit with HS DNA kit (Thermo Fisher).

### Whole exome sequencing (WES)

WES libraries were prepared by the CHOP Center for Applied Genomics (CAG) using the Twist Bioscience Human Core Exome kit with 50 ng of input DNA. Sequencing was performed on the NovaSeq 6000 platform (Illumina) and data were processed using the GATK pipeline [33], as described in the supplemental methods.

### Sanger sequencing

50–100 ng of genomic DNA was used as a template in a 20μl GoTaq (Promega) PCR reaction. The thermal cycling conditions used to generate PCR amplicons for sequencing were: 95 °C initial denaturation for 2 min, 35 cycles of 95 °C for 30 s, 58 °C for 30 s, and 72 °C for 30 seconds, and a final extension of 72 °C for 5 min. The primers used in these PCRs are listed in the supplemental methods. Products were cleaned using a PCR Purification Kit (QIAGEN). Sequencing reactions were commercially performed by Azenta Inc.

### Methylation array

Genomic DNA (500 ng) was bisulfite converted using the EZ DNA Methylation Kit (Zymo Research). Infinium MethylationEPIC array (Illumina) runs were conducted by the CHOP CAG and data was processed using SeSAMe package [34, 35] DMRcate [36, 37] and missMethyl [38–41] R packages as described in the supplemental methods.

### Transcriptome analysis by RNA-Sequencing (RNA-Seq)

Total RNA was isolated from frozen tumor samples using the AllPrep DNA/RNA Micro Kit (QIAGEN). RNA eluate concentration was quantified using the QUBIT HS RNA kit (Thermo Fisher) and quality was assessed by TapeStation (Agilent). The matched BWS non-tumor samples had a low RIN (RNA integrity) value and hence were not included in this library preparation and subsequent analysis. Library preparation of samples meeting appropriate concentration and quality criteria were completed by GENEWIZ, using a Tru-Seq RNA Library Prep kit (Illumina), and included polyA selection. Library quality was assessed by concentration using the QUBIT HS DNA kit (Thermo Fisher) and by fragment size using the TapeStation (Agilent). Sequencing was performed on the Illumina HiSeq 2500 system with 2 ×150 bp read length. Quality of raw reads was assessed using FastQC (https://www.bioinformatics.babraham.ac.uk/projects/fastqc/). Adaptor trimming for Illumina paired-end libraries was applied using Cutadapt [42]. Reads were mapped using STAR [43] to hg19/GRCh37 and count matrices were generated using HTSeq-Count [44].

Read counts were normalized using the variance stabilizing transformation. In the case of comparison with non-syndromic data, the DESeq2 [45] batch correction and apeglm [46] log fold change (LFC) shrinkage was applied to reduce variation between data sets independently sequenced and processed. Gene set enrichment analysis (GSEA) of differentially expressed genes (DEGs) was performed using eVITTA (easy Visualization and Inference Toolbox for Transcriptome Analysis) [47]. Results from GSEA with a with a nominal *p*-value < 0.05 and/or false discovery rate *q*-value < 0.05 were considered enriched. The RNA-Seq network was generated using STRING [48] and hub genes were identified based on nodes with the top 10% of edges. The network with bundled edges to represent interaction counts was generated with the R package edgeBundleR (https://github.com/garthtarr/edgebundleR).

## Results

### Clinical overview of the BWS-WT cohort

Samples analyzed in this study were collected from patients referred for BWS testing at an average age of 35 ±33 months (Table 1). The cohort represents 44% (4/9) females and 55.5% (5/9) of the patients that were diagnosed with BWS-WT (Table 1). At a mean follow-up time of 31 months, 78% (7/9) patients were alive; 29% of these children (2/7) had a relapse event, while 14% (1/7) had metastatic disease (Table 1). Histologically, 78% (7/9) of tumors were classified as favorable and 78% (7/9) with available COG staging were stage 3 or greater (Table 1). Additionally, this study utilized cohorts with as closely matched ages as was feasible.

**Table 1:** Clinical overview of the BWS-WT cohort.

Sample ID	BWS subtype in blood, kidney tumor	Sex	Age at WT diagnosis (months)	Laterality	Histology	Stage	Months off therapy	Outcome	Clinical score
Patient 1 (WT8/NT8)	pUPD11	M	26	Unilateral	Favorable	3	4	Alive	4
Patient 2 (WT9)	pUPD11	M	0	Unilateral	Favorable	Unk	57	Alive	3
Patient 3 (WT10)	pUPD11	M	44	Bilateral	Favorable	5^a^	38	Alive	3
Patient 4 (WT11/NT11)	IC1 GOM	M	45	Unilateral	Unfavorable	4	40	Alive	4
Patient 5 (WT12/NT12)	pUPD11	F	41	Unilateral	Unfavorable	2	37	Alive	3
Patient 6 (WT13/NT13)	IC1 GOM	F	109	Unilateral	Favorable	3	N/A	Deceased	3
Patient 7 (WT14)	pUPD11	M	11	Bilateral	Favorable	5	On therapy	N/A	4
Patient 8 (WT15/NT15)	IC1 GOM	F	34	Bilateral	Favorable	5	N/A	Deceased	2
Patient 9 (WT16/NT16)	pUPD11	F	5	Bilateral	Favorable	5	12	Alive	4

Sample names are retained as per dbGAP submission of the study.

*pUPD11* paternal uniparental disomy of chromosome 11, *IC1 GOM* Imprinting center 1 gain of methylation, *NT* non-tumor, *Unk* unknown.

^a^Unilateral stage 3 each.

### Copy number and single nucleotide variations associated with BWS-WT

**Fig. 1:**
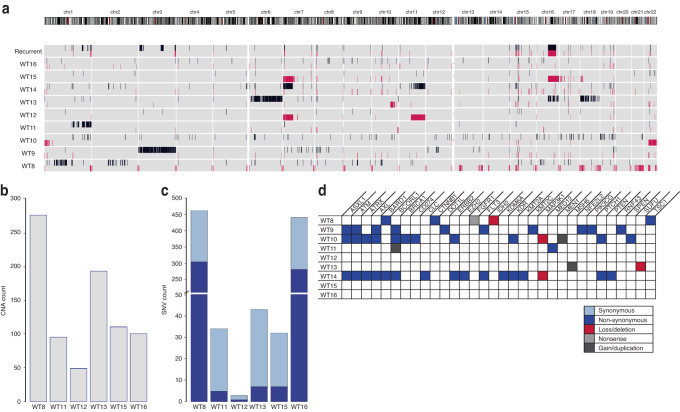
Genomic profile of BWS-WT. **A** Copy number alterations (CNAs) for all BWS-WT. Recurrent changes occur at the same locus in at least two samples. Gains are shown in black; losses are shown in red. **B** CNA for WTs relative to matched normal kidney samples as part of the tumor mutational burden. **C** Single nucleotide variants (SNV) for WTs relative to matched normal kidney samples as part of the tumor mutational burden. **D** CNA and SNV that map to known cancer-driving genes. Red represents loss or deletion; black represents gain or duplication. Light blue represents synonymous SNV, dark blue represents non-synonymous SNV, and gray represents nonsense SNV.

We evaluated copy number alterations (CNAs) and single nucleotide variations (SNVs) using whole exome sequencing. We first assessed BWS-WT for commonly observed alterations seen in nonBWS-WT (including WT1, *TP53, CTNNB1*, LOH at chromosome 16q, content gains at 1q, and content losses at 7p and 17, among others) [6, 49–51]. We also studied recurrent alterations that occurred at the same genomic locations in two or more of the BWS-WT patients. We observed the following recurrent alterations: 100% (9/9) at 15q, 89% (8/9) at 7p, 78% (7/9) at 16q, and 67% (6/9) at 1q, (Fig. 1A, Supplementary Fig. 2A). To determine the tumor mutational burden driving WT in BWS, we normalized the six tumors to their matched non-tumor counterparts, with the goal of determining the number of CNAs in each WT. Using this approach, we found an average of 137 ±78 CNAs per matched set (Fig. 1B). Within BWS WT samples themselves, BWS-WT8 and BWS-WT13 carried a relatively higher number of CNAs, 274 and 192 respectively, but with different distributions. Specifically, BWS-WT13 had many changes on specific chromosomes (chr 6, 16, and 18) and BWS-WT8 presented with chromosome end loss across the genome (Fig. 1A, B).

**Fig. 2:**
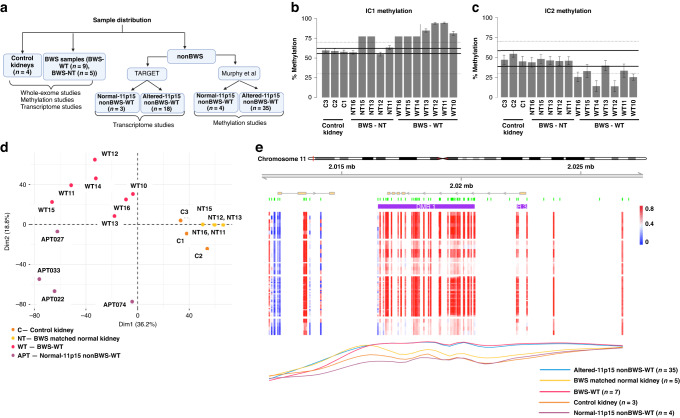
Methylation Status of 11p15 in BWS-WT and nonBWS-WT. **A** Sample groupings based on BWS/nonBWS status plus normal-11p15 vs altered-11p15 nonBWS-WT status used in the study. For IC1 and IC2 methylation results, the area between the dashed lines indicates the normal methylation range presented by Gadd et al. from the TARGET cohort, while the thick black lines indicate the normal range of methylation determined by the control kidney samples used in this study. 11p15 methylation levels for BWS-WT, BWS non-tumor (BWS-NT), and control kidneys (C1, C2, C3) at IC1 (**B**), and IC2 (**C**). **D** Individuals PCA plot of the first two principal components (Dim1 and Dim2) for control kidney (**C**), BWS matched normal kidney (NT), BWS WT (WT), normal-11p15 nonBWS-WT(APT) from Murphy et al. cohort. **E** Differential methylation profile comparing BWS matched normal kidney (*n* = 5), BWS-WT (*n* = 7), altered-11p15 nonBWS-WT (*n* = 35), and normal-11p15 nonBWS-WT(*n* = 4) to control kidneys (*n* = 3) across chromosome 11p15 region. DMR1, the most significant DMR, from both BWS-WT and altered-11p15 nonBWS-WT overlaps the IC1 region on chromosome 11p15.

In our SNV analysis, the six tumors normalized to matched non-tumor counterparts had an average of 85 ±113 SNVs (Fig. 1C). Again, two samples were outliers from the trend; BWS-WT8 and BWS-WT16 each had >400 SNVs (>10/Mb) and over 250 of these were non-synonymous, classifying them as hyper-mutators (Fig. 2C). In the case of BWS-WT8, due to the increased number of SNVs and CNAs localized to chromosome ends, we performed SNV mapping to members of GO:0005697 telomerase holoenzyme complex or GO:0006298 mismatch repair, respectively. Those classified as likely pathogenic or pathogenic/damaging were independently confirmed by Sanger sequencing. Both BWS-NT8 and BWS-WT8 had variants in *TEP1, SMG6*, and *NVL* genes (Supplementary Fig. 2B). Considering BWS-WT16 only had an increased number of SNVs, we explored the possibility of a mismatch repair defect. Both BWS-NT16 and BWS-WT16 had variants in *MLH3* and *MCM9* (Supplementary Fig. 2C).

While these mutations contributed to the genomic landscape of two BWS-WTs, we wanted to identify other putative variants driving WT predisposition and oncogenesis across the cohort. By employing the clinical testing panel of genes used at our institution (which includes 238 genes involved in solid tumor development listed in the Supplemental Methods and is part of our standard of care for the clinical evaluation of WT), we interrogated our WES-identified CNAs and SNVs datasets. Samples without matched non-tumor normalization, BWS-WT9, BWS-WT10, and BWS-WT14, had the largest number of SNVs, but few of these were recurrent among the samples with matched non-tumor WTs (Fig. 1D). The *BCORL1* gene had the highest mutation rate in the cohort (4/9) followed by *ASXL1*, *ATM* and *AXL* genes (3/9; Fig. 1D). All four genes are potential cancer driver genes involved in chromatin remodeling, DNA repair processes, cell cycle processes and Wnt signaling pathways. Interestingly, *CTNNB1* mutation, a driver of WT was observed in one BWS-WT but *TP53, WT1*, but WT-mutated *WTX/AMER1* genes were not observed in any of these BWS-WT (Fig. 1D). In aggregate, the findings from our cohort indicate that the complex mechanisms driving BWS-WT tumorigenesis are largely independent of gene mutations.

### Imprinting of 11p15 in BWS and nonBWS-WT

We next investigated differential methylation profiles through methylation array. The samples in this study were collected from patients diagnosed with BWS due to differential genomic methylation changes (IC1 GOM or pUPD11). We confirmed the molecular changes detected in the clinical lab (Table 1) in BWS WT/matched non-tumor kidney samples by performing differential methylation studies using Infinium MethylationEPIC array. Samples for each analysis are defined in Fig. 2A. We determined the methylation status at 11p15, and the normal methylation range was established as 95% CI (confidence interval) of the control kidney interval, between 55–62% for IC1 and 38–58% for IC2. All BWS-WT samples as well as corresponding BWS non-tumor demonstrated IC1 GOM (Fig. 2B). IC2 LOM was observed in BWS-WT12 and BWS-WT14 (Fig. 2C). As such, these two samples were confirmed as pUPD11, matching their clinical testing designation (Table 1). For BWS-WT10 and BWS-WT16, previous clinical testing identified pUPD11 as indicated in the array results (Fig. 2B, C).

To further define BWS-WT in the context of nonBWS-WT, and expand the power of our cohort, we compared our data with two publicly available non-syndromic/nonBWS cohorts. The nonBWS cohort included the TARGET dataset (*n* = 21) [31]; and Murphy et al. dataset (*n* = 39) [32];. We examined the 11p15 status of the nonBWS tumors in parallel to our samples with normalization to account for batch correction (Supplementary Fig. 1). In the published TARGET WT cohort analysis, a methylation range of 30–70% was considered normal for both IC1 and IC2 and >80% methylation at IC1 was considered as GOM [31]. Applying our narrower normal range, only three TARGET samples demonstrated normal 11p15 methylation at both IC1 and IC2: PAJPHA, PAKKSE, and PALERC (Supplementary Fig. 3A), which were designated as normal-11p15 nonBWS-WT samples (Fig. 2A - which were further used for our WGCNA analysis). Similar evaluation of the Murphy et al. data [32], showed only four samples with normal 11p15 methylation: APT074, APT027, APT022 and APT033 (Supplementary Fig. 3B) which were designated for the differential methylation analysis as normal-11p15 nonBWS-WT samples (Fig. 2A). All other samples from both of these cohorts had aberrant methylation at one or both imprinted regions and were classified as altered-11p15 nonBWS-WT (Supplementary Fig. 3A, B, Fig. 2A).

To evaluate the global methylation profile of BWS and normal-11p15 nonBWS-WT, we performed a principal component analysis (PCA) stratified by 11p15 status on the nonBWS cohort (Murphy et al. data, which used an array platform similar to our dataset) and the BWS-cohort (Supplementary Fig. 3C). In considering the normal-11p15 nonBWS-WT samples from Murphy et al., there was a clear separation of control kidney and BWS non-tumor kidney samples in the first principal component (Dim1), while the WT samples separated along the second principal component (Dim2) according to their methylation status at 11p15 (normal vs altered) (Fig. 2D). This indicates that there are distinct methylation profiles between control kidneys, BWS-NT, BWS-WT samples and normal-11p15 nonBWS-WTs. We also performed PCA on altered-11p15 nonBWS-WT samples and found that these samples segregated from control kidneys and BWS non-tumor kidney samples (Supplementary Fig. 3C). However, the distribution across BWS-WT samples and normal-11p15 nonBWS-WT samples was uneven (Supplementary Fig. 3C). We also applied the methylation range defined by Gadd et al. [31] on Murphy et al. data and found that 2/3 of samples classified as altered-11p15 nonBWS-WT and the other 1/3 as normal-11p15 nonBWS-WT (Supplementary Fig. 3D). The PCA shows that the normal-11p15 nonBWS-WT clustered unevenly as compared to clear separation observed in unsupervised distribution based on our methylation criteria (Supplementary Fig. 3E). This suggests that the methylation range for defining imprinting is narrower in BWS.

Next, we looked for differentially methylated regions (DMRs) between BWS-WT, nonBWS altered-11p15 nonBWS-WT, normal-11p15 nonBWS-WT and control kidneys using the DMRcate package [36, 37]. For both BWS-WT and altered-11p15 nonBWS-WT, the most significant DMR was DMR1, the IC1 region on chromosome 11p15 (chr11:2,016,513–2,020,560) (Fig. 2E) with mean delta β-values of 0.29 and 0.27, respectively. BWS-WT and altered-11p15 nonBWS-WT samples segregated separately from other groups across this region, demonstrating that nonBWS-WT are not the result of undiagnosed BWS. To further highlight the uniqueness of BWS-WT, we performed a region level gene ontology (GO) analysis of the DMRs identified using DMRcate with a mean delta β-value of greater than 15% between WT samples of both cohorts and control kidneys using the missMethyl package [38–41]. The gene ontology analysis was performed in all the groups; BWS-WT, normal-11p15 nonBWS-WT and altered-11p15 nonBWS-WT. Normal-11p15 nonBWS-WT showed enrichment of cellular metabolic processes (Supplementary File 1). BWS-WT showed enrichment of Wnt signaling pathway, integrin signaling pathway, insulin receptor signaling pathway and BMP signaling pathway as shown in Table 2. Altered-11p15 nonBWS-WT showed enrichment of cell cycle processes, DNA damage checkpoint processes, TORC1 signaling, TOR signaling and Wnt signaling pathways as shown in Table 3. These findings demonstrate that differential methylation of Wnt signaling is a common signature of both BWS-WT and altered-11p15 nonBWS-WT, while there are separate unique signals driving WT between study cohorts.

**Table 2:** GO term enrichment of differentially methylated probes in BWS-WT.

	Ontology	Term	Total number of genes in term	Differentially methylated probes	*P*-value	FDR
GO:0198738	BP	Cell-cell signaling by wnt	455	316	9.39E–07	8.45E–05
GO:0016055	BP	Wnt signaling pathway	453	314	1.25E–06	0.00010898
GO:0035567	BP	Non-canonical Wnt signaling pathway	72	58	0.00022179	0.00900615
GO:0007229	BP	Integrin-mediated signaling pathway	113	87	0.00026507	0.01025539
GO:0007219	BP	Notch signaling pathway	181	126	0.0004182	0.01469345
GO:0030111	BP	Regulation of Wnt signaling pathway	333	227	0.00042077	0.01471591
GO:0008286	BP	Insulin receptor signaling pathway	122	88	0.00070441	0.02220243
GO:0046626	BP	Regulation of insulin receptor signaling pathway	72	54	0.0007045	0.02220243
GO:0030510	BP	Regulation of BMP signaling pathway	111	80	0.00117943	0.03309139
GO:0060070	BP	Canonical Wnt signaling pathway	307	206	0.00127743	0.03520494

*BP* Biological Processes.

**Table 3:** GO Term enrichment of differentially methylated probes in altered-11p15 nonBWS-WT.

	Ontology	Term	Total number of genes in term	Differentially methylated probes	*P*-value	FDR
GO:0000075	BP	cell cycle checkpoint signaling	186	119	5.98E–07	4.47E–05
GO:0000077	BP	DNA damage checkpoint signaling	121	80	1.10E–05	0.00066147
GO:0031570	BP	DNA integrity checkpoint signaling	129	84	1.43E–05	0.00083506
GO:1901988	BP	negative regulation of cell cycle phase transition	272	156	1.46E–05	0.00085117
GO:0043067	BP	regulation of programmed cell death	1508	755	9.05E–06	0.00056282
GO:0007093	BP	mitotic cell cycle checkpoint signaling	141	89	3.20E–05	0.0017054
GO:0038202	BP	TORC1 signaling	58	41	0.0001169	0.0054515
GO:0031929	BP	TOR signaling	127	80	0.00012544	0.00576725
GO:0097193	BP	intrinsic apoptotic signaling pathway	298	164	0.00056938	0.0209013
GO:0032006	BP	regulation of TOR signaling	105	65	0.00076791	0.02689792
GO:0016055	BP	Wnt signaling pathway	453	250	0.00114106	0.03708133
GO:0198738	BP	cell-cell signaling by wnt	455	251	0.00118165	0.0382377
GO:0031571	BP	mitotic G1 DNA damage checkpoint signaling	29	22	0.00155439	0.04698605

*BP* Biological Processes.

We also performed gene ontology analysis on samples classified as altered-11p15 nonBWS-WT and normal-11p15 nonBWS-WT based on the methylation range defined by Gadd et al. The normal-11p15 nonBWS-WT cohort showed enrichment of cell cycle processes in addition to cellular metabolic processes (Supplementary File 4) whereas altered-11p15 nonBWS-WT cohort were enriched with GO Terms related to cell cycle, DNA repair, and Wnt signaling among others (Supplementary File 5). The altered-11p15 nonBWS-WT cohort shared signaling features with BWS-WT cohort even though the BWS-WT cohort had a different methylation range for IC1 GOM. This finding demonstrates the importance of defining a narrow methylation range to define BWS-WT, to exclude nonBWS-WT as being miscategorized as a BWS-WT.

### Differential gene expression study

As methylation influences gene expression, we performed differential gene expression analysis between the BWS cohort and control kidneys. Using DEseq2, we obtained a total of 8626 differentially expressed genes (DEGs) with log2fold change >|0.5| and FDR < 0.05. Of these 8626 genes, 48% genes were upregulated, and 52% were downregulated. The list of DEGs is attached as Supplementary File 2.

We first analyzed the expression of the genes subject to genomic imprinting (with parent-specific regulation) due to methylation status at 11p15. We analyzed expression of *H19*, *IGF2*, *KCNQ1*, *KCNQ1OT1* and *CDKN1C*. At IC1, *H19* was similarly expressed between control kidneys and normal-11p15 nonBWS-WT TARGET samples and was downregulated in both altered-11p15 nonBWS-WT TARGET and BWS-WT samples (Supplementary Fig. 4A). *IGF2* was similarly upregulated across all WT samples (Supplementary Fig. 4A). At IC2, *KCNQ1* was downregulated across all WT samples, while *KCNQ1OT1* did not demonstrate significant expression changes between groups (Supplementary Fig. 4A). *CDKN1C* was downregulated in all WT groups compared to control kidneys (Supplementary Fig. 4A). We also examined *WT1* expression at 11p13, as some of the BWS-WT had pUPD11 extend through this region. Expression of *WT1* was upregulated in altered-11p15 nonBWS-WT and BWS-WT as compared to control kidneys and normal-11p15 nonBWS-WT (Supplementary Fig. 4A). Overall, the expression profile of genes subject to genomic imprinting was similar in altered-11p15 nonBWS-WT samples and BWS-WT samples. These expression trends were replicated in the Murphy et al. cohort (Supplementary Fig. 4B), suggesting that changes to genomic imprinting genes are common in WT, regardless of the cause.

**Fig. 3:**
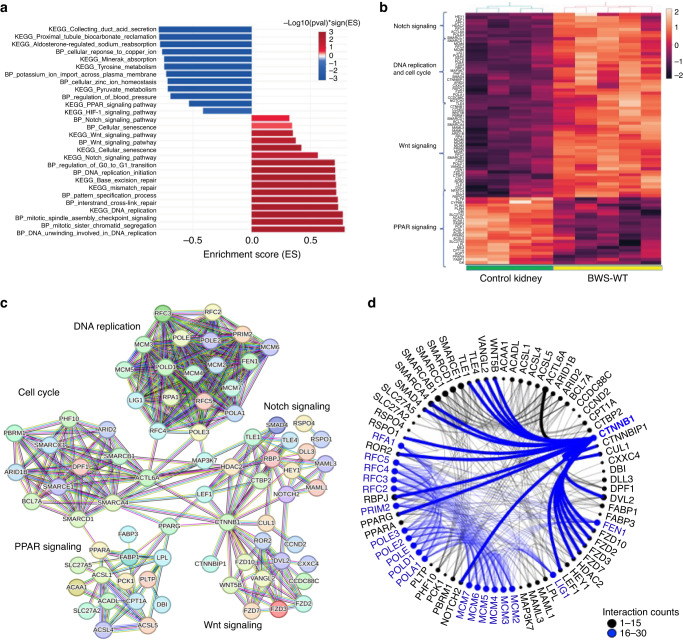
Differential gene expression and interactome analysis. **A** GSEA analysis of DEGs using eVITTA (easy Visualization and Inference Toolbox for Transcriptome Analysis). **B** Heatmap of genes from Wnt signaling pathway, Notch signaling pathway, PPAR signaling pathway, DNA replication and cell cycle processes across BWS-WT and control kidney groups. **C** Protein-protein interaction network generated for genes in heatmap using STRING database. Edges connecting nodes represent functional and physical associations. **D** Interactome was generated using interaction output from STRING analysis which informs most interacting gene partners.

We further performed functional analysis/gene set enrichment analysis (GSEA) with our 8,667 DEGs. The pathways and GO terms that were enriched are shown in Fig. 3A and their enrichment scores and statistical significance are provided in Supplementary File 3. The enriched GO terms included pathways related to both kidney functions and cancer processes. The cancer pathways, including Notch signaling, PPAR signaling, Wnt signaling, DNA replication and cell cycle-related biological processes were enriched in BWS-WT as compared to control kidneys. We observed a clear clustering pattern of genes enriched in these pathways across the control groups and BWS-WT samples (Fig. 3B). We further investigated the protein-protein interaction network of genes enriched in these pathways using STRING database [52]. We observed an intricate network of genes that clustered together in a pathway specific manner (Fig. 3C). We further generated an interactome for these genes using the interaction output from string analysis to understand the gene interaction partners (Fig. 3D). A number of genes including *CTNNB1, FEN1, LIG1, MCM2/3/4/5/6/7, POLA1, POLD1, POLE, POLE2, POLE3, PRM2, RFC2/3/4/5* and *RPA1* showed 16–30 interactions. *CTNNB1* showed the most versatile interactions with genes from different pathways. *CTNNB1* interacted with Wnt signaling pathway genes (*WNT5B, VANGL2, FZD2/3/7/10, CTNNBIP1, LEF1, ROR2, SMAD4*), Notch signaling pathway genes (*NOTCH2, TLE1/4, HDAC2, RBPJ, DVL2, CUL1*), a cell cycle pathway gene *SMARCA4*, a DNA replication pathway gene *RPA1* and a *PPAR* signaling gene *PPARG*. Interestingly, most of these upregulated pathways and interactions are reminiscent of an alteration in the stem/progenitor differentiation programming and nephron patterning [53]. This suggests that BWS-WT may have a dysregulated progenitor cells as a causal agent in disease development and/or progression.

### Weighted gene co-expression network analysis

**Fig. 4:**
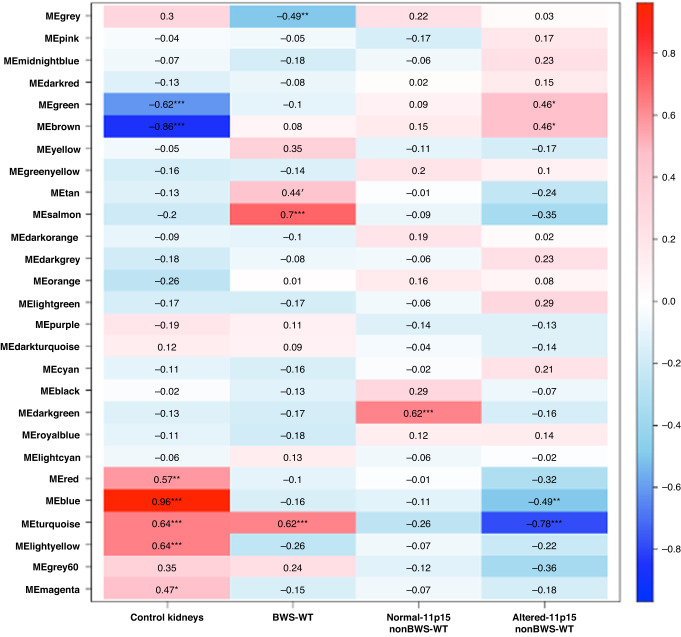
Comparative studies with BWS and nonBWS transcriptome data using WGCNA. Heat map of correlation between the gene modules (MEgrey, MEpink, MEmidnightblue, MEdarkred, MEgreen, MEbrown, MEyellow, MEgreenyellow, MEtan, MEsalmon, MEdarkorange, MEdarkgrey, MEorange, MElightgreen, MEpurple, MEdarkturquoise, MEcyan, MEblack, MEdarkgreen, MEroyalblue, MElightcyan, MEred, MEblue, MEturquoise,MElightyellow, MEgrey60, MEmagenta) and clinical traits of WT patients. The correlation coefficient in each cell represents the correlation between the gene modules and clinical traits, which decreases in size from red to blue. (*p-value < 0.05, **p-value < 0.005, ***p-value < 0.0005). The turquoise, salmon, tan, and gray show significant relations to BWS-WT sample traits; darkgreen showed significant relations to normal-11p15 nonBWS-WT and blue, turquoise showed significant relations to altered-11p15 nonBWS-WT.

We next performed comparative studies with nonBWS data using an unsupervised cluster method - weighted gene co-expression network analysis (WGCNA). We defined four sample traits – control kidneys, BWS-WT, normal-11p15 nonBWS-WT, altered-11p15 nonBWS-WT, as detailed in Supplementary Fig. 1. A total of 27 samples (4 control kidneys, 5 BWS-WT, 3 nonBWS normal-11p15 WT, 15 altered-11p15 nonBWS-WT) were used for this analysis. We used the normalized count obtained from DESeq2 as input for WGCNA. We chose eight as the suitable soft-thresholding power for each set in this analysis based on two criteria described previously [54, 55]. We merged and obtained a total of 27 consensus gene co-expression modules (Supplementary Fig. 5B). The tables of module-trait relationships indicated the relation between the sample traits (control kidneys, BWS-WT, normal-11p15 nonBWS-WT, altered-11p15 nonBWS-WT) and the consensus modules in each data set (Fig. 4). To explain further, turquoise, salmon, tan, and gray showed significant relations to BWS-WT sample traits; darkgreen showed significant relations to normal-11p15 nonBWS-WT and blue, turquoise showed significant relations to altered-11p15 nonBWS-WT. To compare pathways/molecular gene hubs between BWS-WT and nonBWS-WT, we extracted genes associated with those modules-trait partners and subjected them to STRING analysis. Tables 4 and 5 show the enriched terms for both the sample traits BWS-WT and nonBWS-WT in module enriched pattern, respectively. This analysis clearly established the signaling signatures for all the traits in the study. GO terms related to Wnt signaling pathways were enriched in BWS-WT trait as seen by DESeq2 approach for differential gene expression studies; whereas GO terms related to cell cycle processes mark the nonBWS-WT trait. In aggregate, using an unbiased method, we found clear differences existed between BWS-WT, normal-11p15 nonBWS-WT, altered-11p15 nonBWS-WT, and control kidneys, demonstrating that, while some similarities exist between BWS and nonBWS WT, there are clear distinctions between the groups.

**Table 4:** GO Term enrichment analysis for BWS-WT modules.

MEgrey					
#term ID	Term description	Observed gene count	Background gene count	Strength	False discovery rate
GO:0006119	Oxidative phosphorylation	22	118	0.56	0.0156
GO:0046034	ATP metabolic process	29	204	0.44	0.0243
GO:0022904	Respiratory electron transport chain	19	107	0.54	0.0483
GO:0042775	Mitochondrial atp synthesis coupled electron transport	17	87	0.58	0.0483
**MEtan**					
GO:0000398	mRNA splicing, via spliceosome	20	294	0.54	Not significant
**MEsalmon**					
GO:0006521	Regulation of cellular amino acid metabolic process	12	64	0.67	0.0268
GO:0061418	Regulation of transcription from rna polymerase ii promoter in response to hypoxia	14	78	0.65	0.0178
GO:0060071	Wnt signaling pathway, planar cell polarity pathway	17	96	0.64	0.0101
GO:1902036	Regulation of hematopoietic stem cell differentiation	13	74	0.64	0.0255
GO:0038061	NIK/NF-kappaB signaling	14	81	0.63	0.0216
GO:0090175	Regulation of establishment of planar polarity	18	111	0.61	0.0101
GO:0033209	Tumor necrosis factor-mediated signaling pathway	20	125	0.6	0.0101
GO:0031145	Anaphase-promoting complex-dependent catabolic process	13	83	0.59	0.0445
GO:0010972	Negative regulation of g2/m transition of mitotic cell cycle	14	92	0.58	0.0364
GO:0070498	interleukin-1-mediated signaling pathway	14	96	0.56	0.0445
GO:0035567	Non-canonical wnt signaling pathway	18	130	0.54	0.0216
GO:0038095	Fc-epsilon receptor signaling pathway	16	115	0.54	0.0325

**Table 5:** GO term enrichment analysis for altered-11p15 nonBWS-WT modules.

MEturquoise					
#term ID	Term description	Observed gene count	Background gene count	Strength	False discovery rate
GO:0090307	Mitotic spindle assembly	14	43	0.61	0.02
GO:0007052	Mitotic spindle organization	22	81	0.53	0.0061
GO:0051225	Spindle assembly	21	88	0.47	0.0199
GO:0007051	Spindle organization	33	145	0.45	0.0024
GO:1902850	Microtubule cytoskeleton organization involved in mitosis	24	112	0.43	0.0214
GO:0140014	Mitotic nuclear division	30	156	0.38	0.0199
**MEgreen**					
GO:0071704	Organic substance metabolic process	486	7755	0.08	0.0011
GO:0044238	Primary metabolic process	459	7332	0.08	0.0023
GO:0044237	Cellular metabolic process	467	7513	0.08	0.0025
GO:0008152	Metabolic process	504	8298	0.07	0.0074
GO:0000278	Mitotic cell cycle	70	695	0.26	0.0499
GO:1903047	Mitotic cell cycle process	63	616	0.27	0.0499
**MEbrown**					
GO:0043603	Cellular amide metabolic process	84	773	0.24	0.0257
GO:0000278	Mitotic cell cycle	76	695	0.25	0.0323
GO:0034641	Cellular nitrogen compound metabolic process	264	3282	0.11	0.0323
GO:1903047	Mitotic cell cycle process	68	616	0.25	0.0385

## Discussion

Patients with BWS have an increased risk for developing WT, especially those with IC1 GOM and pUPD11 [56, 57]. Even in the non-syndromic population, at least two-thirds of WT demonstrate these BWS-like alterations across 11p15 [5, 19, 22]. While this association is well-established, this is the first study examining the molecular mechanisms underlying the link between BWS and WT oncogenesis based on 11p15 status. In this study, we present the first molecular multi-omics study of a cohort of BWS-WT. Our results highlight the similarities and differences between these altered-11p15 nonBWS-WT and BWS-WT tumors.

Epigenetic and genomic changes, aside from those at 11p15, have been considered for their prognostic and molecular contribution to WT oncogenesis. While mutation frequencies are generally lower in pediatric tumors compared to adult tumors, in our cohort, two of our BWS patients (BWS-WT8, BWS-WT16) exhibited high mutation frequencies [58]. BWS-NT8, BWS-WT8 had variants in *TEP1, SMG6*, and *NVL* genes related to telomerase activity and BWS-NT16, BWS-WT16 had variants in *MLH3* and *MCM9* gene related to DNA mismatch repair. However, further studies are required to consider their specific contributions to BWS-WT oncogenesis, given that only a subset of our cohort carried these changes. While mutations in the cancer driver genes *WT1, TP53*, and *CTNNB1* are common and even co-occur in WT [9, 19, 29, 59], syndromic or sporadic tumors with 11p15 alterations are less affected by these mutations [9, 25, 60]. In this cohort of BWS-WT, we did not observe *WT1* or *TP53* mutations in any samples and only one sample exhibited a mutation in *CTNNB1*. Our BWS-WT cohort showed highest mutation rate in *BCORL1* followed by *ASXL1, ATM* and *AXL* genes. *BCORL1* and *ASXL1* mutations are mainly seen in hematologic malignancies and aplastic anemia [61, 62]. *BCORL1* is a homolog of the transcription factor BCOR (BCL6 corepressor) gene and a subunit of polycomb repressive complex 1 (PRC1) complex – a major chromatin remodeler [63]. Damm et al. reported that BCOR mutations arise after mutations affecting genes involved in splicing machinery or epigenetic regulation [64]. This suggests *BCORL1* mutations might be associated with epigenetic changes seen in BWS. It has been previously suggested that 11p15 changes are early clonal events [22, 49, 65, 66]; it is possible that 11p15-associated overgrowth is sufficient to jumpstart WT oncogenesis and does not require specific mutations in *WT1, TP53 or CTNNB1*. Previously, we have shown that 11p15 alterations dysregulate cell cycle restriction in BWS non-tumor liver [67].

We defined a narrow methylation range for IC1 and IC2 based on our patient cohort and expertise in defining low-level mosaicism in BWS [30]. This narrow range was sufficient to stratify publicly available datasets with non-BWS normal and altered-11p15 nonBWS-WT. We classified very few samples as normal-11p15 nonBWS-WT highlighting the important role of 11p15 epigenetics in driving WT oncogenesis. After performing this stratification, we studied the methylation pattern of these samples across the genome. The PCA of DMRs clearly showed that our stratification was effective in separating normal-11p15 nonBWS-WT samples from other groups in study. The DMR study using the methylation data showed that the most significant DMR was on chromosome 11p15 corresponding to IC1. This finding demonstrates the importance of the 11p15 region in global gene regulation. A previous clinical report supports the WT risk associated with constitutional abnormalities at the imprinted 11p15 growth regulatory region [68].

The gene ontology study of DMRs showed that the Wnt signaling pathway played a significant role in BWS-WT and altered-11p15 nonBWS-WT oncogenesis. A recent study on WT by Brzezinski et al. also showed differential methylation of genes in the Wnt signaling pathway [29]. Thus, differential methylation of genes has a major role to play in WT oncogenesis [69]. This observation was further validated by transcriptome studies. BWS-WT showed dysregulation of Wnt signaling along with other distinct pathways including Notch signaling, BMP signaling, PPAR signaling and, NIK/NF-kappaB signaling pathways. These findings are in line with a potential defect in stem/progenitor cell biological processes and nephron patterning [53], which may drive BWS-WT initiation and/or tumor survival. WT is embryonic-derived tumor [70] resembling fetal kidney with disorganized nephrogenic structures [71]. Since WT development is tightly linked to its developmental process [71], understanding of tissue-residing progenitor cell regulation is imperative. By studying signaling pathways, we provide a mechanistic rationale for molecular mechanisms of WT oncogenesis that comprise dysregulation of nephron progenitor cells in WT. However, a dedicated model is required to explain the combinatorial role of these pathways in the earliest events of WT tumorigenesis.

Both methylation and transcriptome data showed that cell cycle and DNA replication-related processes are shared features for BWS-WT and altered-11p15 nonBWS-WT, which is supported by other studies as well [72, 73]. We also studied the protein-protein interaction network of the genes in the BWS-WT oncogenesis pathway. The generated interactome from this protein network showed that *CTNNB1*, a major Wnt signaling molecule, had the most interactions with genes from different pathways. There are numerous reports on *CTNNB1* mutations in sporadic WT [9, 74–76]. These studies indicate that *CTNNB1* and other mutations, underlie the genetic basis for WT oncogenesis. However, in our cohort of BWS-WT, only one patient carried a *CTNNB1* mutation. Overall, we observed upregulation of wildtype *CTNNB1* in BWS-WT, with broadest range of CTNNB1 interactions with genes across other signaling pathways. This observation suggests that BWS-WT has a unique signature of Wnt signaling driven by *CTNNB1* overexpression which also has a major role in nephron patterning [53]. Further work will be required to understand the implication of *CTNNB1* overexpression and activity in BWS-WT oncogenesis.

**Fig. 5:**
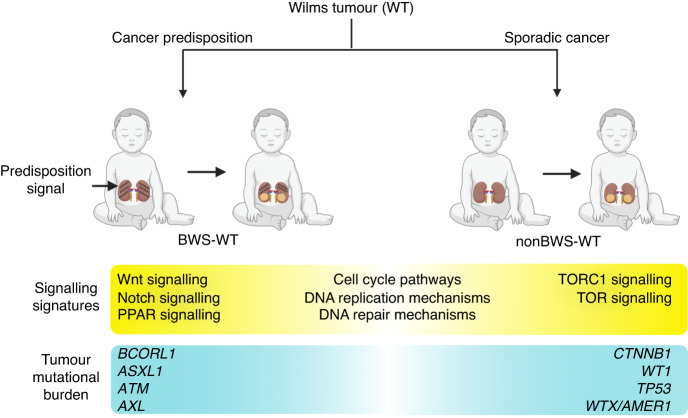
Spectrum of molecular signaling pathways in WT oncogenesis. Both BWS-WT and altered-11p15 nonBWS-WT show enrichment of cell cycle pathways, DNA replication mechanisms and DNA repair mechanisms in addition to unique molecular pathways. Both BWS-WT and altered-11p15 nonBWS-WT exhibit unique SNVs.

WGCNA, a scale-free network distribution approach, is powerful data-driven tool to study gene expression pattern in samples from different cohorts [77]. Using WGCNA, we were able to validate the DEG pathway signatures in BWS-WT. We identified 27 modules, most related to BWS-WT showing enrichment of the Wnt, NIK/NF-kappaB, cell cycle, and TNF-mediated signaling pathways. Other modules were enriched for regulatory/metabolic processes. Modules enriched for altered-11p15 nonBWS-WT trait mainly showed enrichment of cell cycle and metabolic processes. Many other studies on WT using the WGCNA approach have showed enrichment of cell cycle related processes, indicating it as a major molecular signature for WT [77–79]. Along with metabolic pathway enrichment, Wang et al. have also shown enrichment of PI3K-Akt, FoxO, p53, and TNF signaling pathways, along with many other cancer related pathways in WT, using the WGCNA approach [78]. This indicates that altered-11p15 nonBWS-WT and BWS-WT have some common features but also have unique gene signatures attributed to the epigenetic predisposition in BWS. WT oncogenesis can be seen as spectrum of molecular signaling signatures and differential tumor mutational burden (Fig. 5) seen in BWS and nonBWS cohorts. This is the first study that highlights these features on WT oncogenesis based on 11p15 alterations.

However, there are some limitations in this study. A major limitation is its sample size due to the rarity of this syndrome and sample type. When possible, we have included data from the TARGET and Murphy cohorts to increase the robustness of our study. Additionally, while some patient-specific variation existed in our cohort, the main findings were supported across the entirety of the cohort, suggesting that our findings would be comparable to a larger study. Another limitation is the lack of matched normal kidney samples for RNA-Seq, as we were unable to generate the required cDNA sequencing libraries, since the patients were subjected to chemotherapy and the quality of the samples was poor. Also, the publicly available cohort did not have matched normal kidney samples. However, by combining different approaches, including whole exome sequencing, methylation, and transcriptome studies, we successfully captured the intricate molecular signaling that drives WT oncogenesis. Our identification of dysregulated signaling pathways for cell differentiation, growth-promotion, and cell cycle regulation require additional evaluation in larger cohorts for their application as diagnostic or therapeutic targets in patient WT oncogenesis. However, we have successfully stratified WT based on 11p15 alterations, which will be the subjects of future mechanistic studies in patient-derived tissues, as well as for early stage in vitro screening of therapeutic drugs.

### Supplementary information

Supplementary Methods and figures

Supplementary File 1

Supplementary File 2

Supplementary File 3

Supplementary File 4

Supplementary File 5

## Data Availability

Data generated in this study have been deposited in the Database of Genotypes and Phenotypes (dbGAP) of the National Center for Biotechnology Information (United States National Library of Medicine, Bethesda, MD) under accession number phs002769.v1.p1.
