# Supplementary material for: Cancer predisposition signaling in Beckwith-Wiedemann Syndrome drives Wilms tumor development
Source: Br J Cancer. 2023 Dec 23;130(4):638–50. doi: 10.1038/s41416-023-02538-x (PMC10876704; doi:10.1038/s41416-023-02538-x)
Supplement: Supplementary file 1 — Supplementary Methods and figures [file 41416_2023_2538_MOESM1_ESM.docx]

**Title**

Cancer Predisposition Signaling in Beckwith-Wiedemann Syndrome Drives Wilms Tumor Development

**Authors**

Snehal Nirgude, Natali S. Sobel Naveh, Sanam L. Kavari, Emily M. Traxler, Jennifer M. Kalish

**Supplemental Methods**

Genomic DNA analysis

*Whole Exome Sequencing*

Human Core Exome and Human RefSeq Panel (Twist Bioscience) exon capture probes were used in 8-plex pools and the libraries were prepared on the Sciclone robot (Perkin Elmer). Quality of libraries, i.e. fragment size and concentration, was determined on the GX platform (Perkin Elmer). Sequencing was performed on the NovaSeq 6000 platform (Illumina) on an S2 200 cycle v1.0 flow cell with dual index paired end 2x100 bp run parameter. Raw data was deduplexed using Dragen (Illumina).

The exome data was processed using the GATK pipeline (1) as follows: unmapped .bam files were generated using PICARD while mapped .bam files were generated using BWA-MEM to align reads to hg19/GRC37. Sorting and merging of .bam files were performed using Samtools (2), PCR duplicates were marked, and base recalibration was applied using PICARD (<http://broadinstitute.github.io/picard>). Single nucleotides variants (SNVs) were identified using GATK Mutect2 in paired tumor sample or single tumor sample mode, false positives were filtered, and passing SNVs were summarized using Annovar (3). The tumor mutational burden was estimated by comparing variant calls to liver controls from within the study as well as 30 (>3x) unrelated non-cancer exomes produced on the same platform through the CHOP CAG. SNV classification was derived from the refGene exonic function column. Likely pathogenic/damaging or pathogenic/damaging variants were identified based on agreement of at least SIFT and PolyPhen2 algorithms (4, 5). Copy number alterations (CNAs) were called using the R package ExomeDepth (6). Recurrent CNAs were identified by the R package CNVranger (7) using p-value < 0.05 for alterations occurring in at least two samples. Visualization of CNA analysis was achieved using the R package CopyNumberPlots (<https://github.com/bernatgel/CopyNumberPlots>). For both SNVs and CNAs, results were filtered based on gene names derived from the RNA-Seq analysis component as well as 238 known to be associated with a variety of pediatric solid tumors that are included in the CHOP clinical solid tumor panel test: *ABL1, ACVR1, AKT1, AKT2, AKT3, ALK, AMER1, APC, AR, ARAF, ARID1A, ARID1B, ARID2, ASXL1, ATM, ATR, ATRX, AURKA, AURKB, AXIN1, AXL, B2M, BAP1, BARD1, BCL2, BCL6, BCOR, BCORL1, BLM, BRAF, BRCA1, BCRA2, BRD4, BRIP1, CARD11, CBFB, CBL, CCND1, CCND2, CCND3, CCNE1, CD274, CD79B, CDC73, CDH1, CDK12, CDK4, CDK6, CDK8, CDKN1B, CDKN2A, CDKN2B, CDKN2C, CHEK1, CHEK2, CIC, CREBBP, CRKL, CRLF2, CSF1R, CTCF, CTNNB1, DAXX, DDR2, DICER1, DNMT3A, DOT1L, EED, EGFR, EP300, EPHA3, EPHA5, EPHB1, ERBB2, ERBB3, ERBB4, ERG, ESR1, ETV6, EZH2, FAM46C, FANCA, FANCC, FBXW7, FGF19, FGF3, FGF4, FGFR1, FGFR2, FGFR3, FGFR4, FLCN, FLT1, FLT3, FLT4, FOXL2, FOXP1, FUBP1, GATA1, GATA2, GATA3, GNA11, GNAQ, GNAS, GRIN2A, GSK3B, H3F3A, HGF, HIST1H1C, HIST1H3B, HNF1A, HRAS, IDH1, IDH2, IGF1R, IKBKE, IKZF1, IL7R, INPP4B, IRF4, IRS2, JAK1, JAK2, JAK3, JMJD1C, JUN, KDM5A, KDM5C, KDM6A, KDR, KEAP1, KIT, KMT2A, KMT2C, KRAS, MAP2K1, MAP2K2, MAP2K4, MAP3K1, MAPK1, MCL1, MDM2, MDM4, MED12, MEF2B, MEN1, MET, MITF, MLH1, MPL, MRE11A, MSH2, MSH6, MTOR, MUTYH, MYB, MYC, MYCN, MYD88, MYOD1, NF1, NF2, NFE2L2, NKX2-1, NOTCH1, NOTCH2, NPM1, NRAS, NTRK1, NTRK2, NTRK3, PALB2, PAX5, PBRM1, PDCD1, PDGFRA, PDGFRB, PHOX2B, PIK3CA, PIK3CG, PIK3R1, PIK3R2, PIM1, PPM1D, PPP2R1A, PRDM1, PRKAR1A, PTCH1, PTEN, PTPN11, RAD50, RAD51, RAF1, RARA, RB1, RET, RHOA, RICTOR, RNF43, ROS1, RPTOR, RUNX1, SDHA, SDHB, SDHC, SDHD, SETD2, SF3B1, SMAD2, SMAD4, SMARCA4, SMARCB1, SMO, SOCS1, SOX2, SPEN, SPOP, SRC, STAG2, STK11, SUFU, SUZ12, TERT, TET2, TGFBR2, TNFAIP3, TNFRSF14, TOP1, TP53, TP63, TSC1, TSC2, TSHR, U2AF1, VHL, WHSC1, WT1, XPO1.*

*Sanger Sequencing*

Primers used in these PCRs and subsequent sequencing reactions are as follows:
rs65685_F 5-CCTTCTCTCTTGGTCCGTGT-3,
rs65685_R 5-CATGGAGGGCAGACAATGTG-3,
rs1760903_F 5-AGGGGTATCTGTGGGCAAAT-3,
rs1760903_R 5-GCTCTCATCTACTTCCGGGA-3,
rs1885986_F 5-GGCTCATCAATTCTGTCCTCG-3,
 rs1885986_R 5-AGTAGGGGCTGCAAAAGGAG-3,
rs28756986_F 5-ACGGACGATTGGTTTGGAGA-3,
rs28756986_R 5- ACTTTGAGGAGAGTGGGCAG-3,
rs34631151_F 5-TCCCTGGATGCTTCATCTGG-3,
rs34631151_R 5- ATGCTTGACCCTCTGTGCTT-3.

*Methylation Array*

Two separate reactions of 500 ng of genomic DNA were converted for each sample using the incubation recommended by the manufacturer for use with Illumina arrays. Eluates were pooled and quantified using the Nanodrop (Thermo Fisher) RNA assay. Approximately 600 ng of converted DNA was used for each array reaction. Genomic DNA from BWS WT8, NT8, and WT9 were not of suitable quality and concentration for running on the methylation array, so it was not included in this analysis.

For Murphy cohort and BWS cohort, raw intensity files were processed in R using the SeSAMe package (8, 9). Raw intensity files were converted to Beta values using the openSesame pipeline which includes a quality mask for probes of poor design, a non-linear dye bias correction, background subtraction using out-of-band probes, and masking based on detection p-value of out-of-band probes. Wilms tumor samples from Murphy et al. 2019 (10) were stratified based on their methylation status at the imprinting control regions on chromosome 11p15. Those with loss of methylation at the KvDMR1/IC2 (GRCh37/hg19 chr11:2720228-2722714) and/or gain of methylation at the H19-DMR/IC1 (GRCh37/hg19 chr11:2019078-2024127) relative to control kidney samples were defined as altered at 11p15, while tumors with normal methylation at both sites relative to control kidney were defined as normal at 11p15. BWS Wilms tumor, altered 11p15 Wilms tumor, and normal 11p15 Wilms tumor samples were compared to control kidney samples to identify differentially methylated regions (DMRs) using DMRcate (11, 12) followed by gene ontology analysis of DMRs using the goregion function in the missMethyl package (13-16).

For TARGET cohort, raw intensity files were processed in R using the methylationArrayAnalysis (<https://www.bioconductor.org/packages/release/workflows/html/methylationArrayAnalysis.html>) package. Samples were assessed for overall probe call quality before inclusion. Probes were assessed for single nucleotide polymorphisms; those that overlapped such sites were removed from the analysis. Tumor samples were compared to matched non-tumor samples to identify differentially methylated probes, regions, and pathways. Significant methylation differences were identified using ChAMP(17, 18). Methylation levels at 11p15 were established based on the range represented by the three controls analyzed in this study. For all cohorts, methylation levels at 11p15 were established based on the range represented by the three controls analyzed in this study. Samples with confidence intervals that touch or overlap with this range were considered “normal-11p15”, while samples with confidence intervals outside of this range were considered to demonstrate “aberrant-11p15” methylation.

**
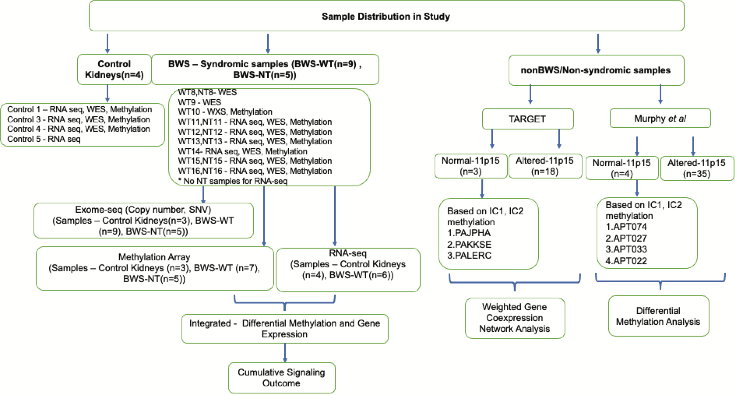
**

**Supplementary Figure 1**: **Summary of Sample distribution in the study**. BWS – Beckwith Wiedemann Syndrome, WT- Wilms tumor, NT- non-tumor, SNV- single nucleotide variant, IC1 – imprinting center 1, IC2- imprinting center 2, WES- Whole exome sequencing.

**Supplementary Figure 2. Specific genome features of BWS WT. (A)** Single nucleotide variants (SNVs) captured by Whole Exome Sequencing are visualized using Integrated Genome Viewer (19) in genome build hg19 on chromosome 16. For BWS WT11, 12, 13, and 16, informative SNVs indicate no loss-of-heterozygosity. **(B)** Sanger sequencing electropherograms show telomerase-associated pathogenic or likely pathogenic variants in *TEP1, SMG6,* and *NVL* genes in BWS non-tumor (NT) and WT8. **(C)** Sanger sequencing electropherograms show mismatch repair-associated pathogenic or likely pathogenic variants in *MLH3* and *MCM9* in BWS NT and WT16.

**Supplementary Figure 3.** 11p15 methylation status in the non-syndromic WT. For IC1 and IC2 methylation results, the area between the dashed lines indicates the normal methylation range presented by Gadd et al., while the thick lines indicate the normal range of methylation determined by the control kidney samples used in this study. Error bars indicate the 95% confidence intervals. **(A)** TARGET non-syndromic WT cohort 11p15 methylation levels. **(B)** Murphy *et al*. 2019 non-syndromic WT cohort methylation levels. **(C)** PCA plot for all the samples in the differential methylation study. **(D)** Summary of Sample distribution in the study (BWS – Beckwith Wiedemann Syndrome, WT- Wilms tumor, NT- non-tumor, SNV- single nucleotide variant, IC1 – imprinting centre 1, IC2- imprinting center 2, WES- Whole exome sequencing) with methylation range defined from Gadd et al. study for nonBWS cohort **(20)**. **(E)** Individuals PCA plot of the first two principal components (Dim1 and Dim2) for Control kidney (C), BWS matched normal kidney (NT), BWS WT (WT), normal-11p15 nonBWS-WT (APT).

**Supplementary Figure 4**: **(A)** Expression profile of imprinted genes for TARGET non-syndromic (NS) WT cohort in comparison to BWS cohort. **(B)** Expression profile of imprinted genes for Murphy *et al*. non-syndromic WT cohort.

**Supplementary Figure 5**: **(A)** The soft-thresholding power was defined using the scale free topology fit index (y-axis; top) and the mean connectivity (y-axis; bottom) under different power (x-axis). **(B)** Hierarchical clustering dendrogram of identified co‑expressed genes in modules by WGCNA. The dendrogram shows the clustering tree of genes (top), primary unmerged modules (middle) and merged modules (bottom). The unmerged colored strips in middle indicate the module designation identified though the clusters of co‑expressed genes and assigned the merged module color to the original module color.

**Supplemental References**

1. Geraldine A. Van der Auwera BDOC. Genomics in the Cloud: O'Reilly Media, Inc.; 2020.

2. Bonfield JK, Marshall J, Danecek P, Li H, Ohan V, Whitwham A, et al. HTSlib: C library for reading/writing high-throughput sequencing data. Gigascience. 2021;10(2).

3. Wang K, Li M, Hakonarson H. ANNOVAR: functional annotation of genetic variants from high-throughput sequencing data. Nucleic Acids Res. 2010;38(16):e164.

4. Adzhubei I, Jordan DM, Sunyaev SR. Predicting functional effect of human missense mutations using PolyPhen-2. Curr Protoc Hum Genet. 2013;Chapter 7:Unit7 20.

5. Ng PC, Henikoff S. SIFT: Predicting amino acid changes that affect protein function. Nucleic Acids Res. 2003;31(13):3812-4.

6. Plagnol V, Curtis J, Epstein M, Mok KY, Stebbings E, Grigoriadou S, et al. A robust model for read count data in exome sequencing experiments and implications for copy number variant calling. Bioinformatics. 2012;28(21):2747-54.

7. da Silva V, Ramos M, Groenen M, Crooijmans R, Johansson A, Regitano L, et al. CNVRanger: association analysis of CNVs with gene expression and quantitative phenotypes. Bioinformatics. 2020;36(3):972-3.

8. Zhou W, Triche TJ, Jr., Laird PW, Shen H. SeSAMe: reducing artifactual detection of DNA methylation by Infinium BeadChips in genomic deletions. Nucleic Acids Res. 2018;46(20):e123.

9. Triche TJ, Jr., Weisenberger DJ, Van Den Berg D, Laird PW, Siegmund KD. Low-level processing of Illumina Infinium DNA Methylation BeadArrays. Nucleic Acids Res. 2013;41(7):e90.

10. Murphy AJ, Chen X, Pinto EM, Williams JS, Clay MR, Pounds SB, et al. Forty-five patient-derived xenografts capture the clinical and biological heterogeneity of Wilms tumor. Nat Commun. 2019;10(1):5806.

11. Peters TJ, Buckley MJ, Statham AL, Pidsley R, Samaras K, R VL, et al. De novo identification of differentially methylated regions in the human genome. Epigenetics Chromatin. 2015;8:6.

12. Peters TJ, Buckley MJ, Chen Y, Smyth GK, Goodnow CC, Clark SJ. Calling differentially methylated regions from whole genome bisulphite sequencing with DMRcate. Nucleic Acids Res. 2021;49(19):e109.

13. Maksimovic J, Gordon L, Oshlack A. SWAN: Subset-quantile within array normalization for illumina infinium HumanMethylation450 BeadChips. Genome Biol. 2012;13(6):R44.

14. Phipson B, Oshlack A. DiffVar: a new method for detecting differential variability with application to methylation in cancer and aging. Genome Biol. 2014;15(9):465.

15. Maksimovic J, Gagnon-Bartsch JA, Speed TP, Oshlack A. Removing unwanted variation in a differential methylation analysis of Illumina HumanMethylation450 array data. Nucleic Acids Res. 2015;43(16):e106.

16. Phipson B, Maksimovic J, Oshlack A. missMethyl: an R package for analyzing data from Illumina's HumanMethylation450 platform. Bioinformatics. 2016;32(2):286-8.

17. Morris TJ, Butcher LM, Feber A, Teschendorff AE, Chakravarthy AR, Wojdacz TK, et al. ChAMP: 450k Chip Analysis Methylation Pipeline. Bioinformatics. 2014;30(3):428-30.

18. Tian Y, Morris TJ, Webster AP, Yang Z, Beck S, Feber A, et al. ChAMP: updated methylation analysis pipeline for Illumina BeadChips. Bioinformatics. 2017;33(24):3982-4.

19. Robinson JT, Thorvaldsdottir H, Wenger AM, Zehir A, Mesirov JP. Variant Review with the Integrative Genomics Viewer. Cancer Res. 2017;77(21):e31-e4.

20. Gadd S, Huff V, Walz AL, Ooms A, Armstrong AE, Gerhard DS, et al. A Children's Oncology Group and TARGET initiative exploring the genetic landscape of Wilms tumor. Nat Genet. 2017;49(10):1487-94.
